# Etymologia: *Neisseria*

**DOI:** 10.3201/eid2206.ET2206

**Published:** 2016-06

**Keywords:** etymologia, Neisseria gonorrhoeae, Neisseria meningitidis, Diplococcus intracellularis meningitidis, bacteria, gonorrhea, meningitis, septicemia, Albert Neisser, Anton Weichselbaum

## *Neisseria* **[**ni-seʹre-ə**]**

A gram-negative, non-motile diplococcal bacterium, *Neisseria* (Figure) is named after Albert Ludwig Sigesmund Neisser, a German physician who discovered *Neisseria gonorrhoeae* in 1879.

**Figure:**
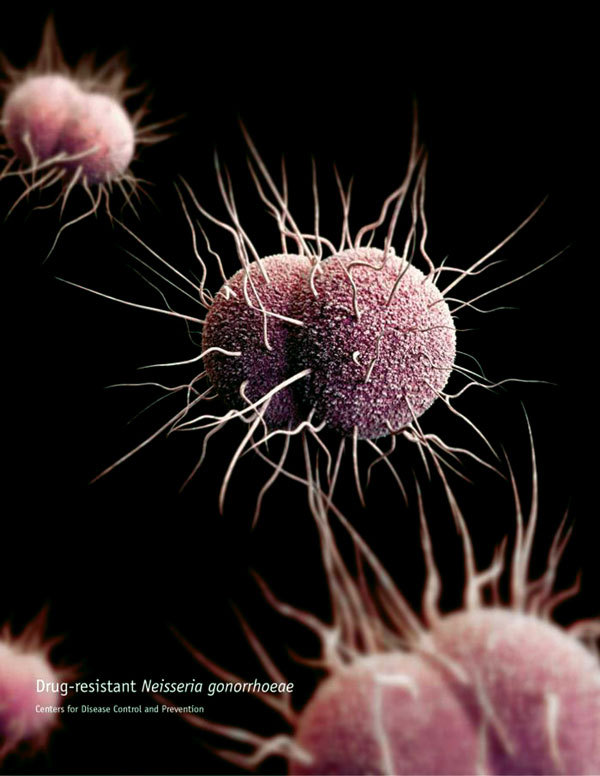
A 3-dimensional computer-generated image of drug-resistant Neisseria gonorrhoeae diplococcal bacteria. Source: Public Health Image Library.

Gonorrhea comes from the Greek *gonos*, meaning “seed,” and *rhoe*, “flow. The disease caused by this bacterium was known as “gonorrhea” because early physicians incorrectly thought the purulent discharge was semen. As early as 1719, gonorrhea was referred to as “the clap,” although theories for why it was called this vary. It may refer to the old French term *clapier*, “brothel,” a place where the disease spread easily. Another theory refers to preantibiotic days when the infection was treated by slapping the penis against a board, or clapping it between two boards to force out infected discharge.

*N. gonorrhoeae* is 1 of only 2 *Neisseria* species that is pathogenic to humans. The second, *N. meningitidis*, causes outbreaks of meningitis and septicemia. It was isolated by Anton Weichselbaum in 1887 and designated as *Diplococcus intracellularis meningitidis*.
